# The impact of blood type O on mortality of severe trauma patients: a retrospective observational study

**DOI:** 10.1186/s13054-018-2022-0

**Published:** 2018-05-02

**Authors:** Wataru Takayama, Akira Endo, Hazuki Koguchi, Momoko Sugimoto, Kiyoshi Murata, Yasuhiro Otomo

**Affiliations:** 1 Trauma and Acute Critical Care Medical Center, Tokyo Medical and Dental University Hospital of Medicine, 1-5-45, Yushima, Bunkyo-ku, Tokyo, 113-0034 Japan; 2 The Shock Trauma and Emergency Medical Center, Matsudo City Hospital, 4005, Kamihongo, Matsudo, Chiba Japan

**Keywords:** ABO blood type, Bleeding, Trauma, Exsanguination, Von Willebrand factor

## Abstract

**Background:**

Recent studies have implicated the differences in the ABO blood system as a potential risk for various diseases, including hemostatic disorders and hemorrhage. In this study, we evaluated the impact of the difference in the ABO blood type on mortality in patients with severe trauma.

**Methods:**

A retrospective observational study was conducted in two tertiary emergency critical care medical centers in Japan. Patients with trauma with an Injury Severity Score (ISS) > 15 were included. The association between the different blood types (type O versus other blood types) and the outcomes of all-cause mortality, cause-specific mortalities (exsanguination, traumatic brain injury, and others), ventilator-free days (VFD), and total transfusion volume were evaluated using univariate and multivariate competing-risk regression models. Moreover, the impact of blood type O on the outcomes was assessed using regression coefficients in the multivariate analysis adjusted for age, ISS, and the Revised Trauma Score (RTS).

**Results:**

A total of 901 patients were included in this study. The study population was divided based on the ABO blood type: type O, 284 (32%); type A, 285 (32%); type B, 209 (23%); and type AB, 123 (13%). Blood type O was associated with high mortality (28% in patients with blood type O versus 11% in patients with other blood types; *p* <  0.001). Moreover, this association was observed in a multivariate model (adjusted odds ratio = 2.86, 95% confidence interval 1.84–4.46; *p* <  0.001). The impact of blood type O on all-cause in-hospital mortality was comparable to 12 increases in the ISS, 1.5 decreases in the RTS, and 26 increases in age. Furthermore, blood type O was significantly associated with higher cause-specific mortalities and shorter VFD compared with the other blood types; however, a significant difference was not observed in the transfusion volume between the two groups.

**Conclusions:**

Blood type O was significantly associated with high mortality in severe trauma patients and might have a great impact on outcomes. Further studies elucidating the mechanism underlying this association are warranted to develop the appropriate intervention.

**Electronic supplementary material:**

The online version of this article (10.1186/s13054-018-2022-0) contains supplementary material, which is available to authorized users.

## Background

The ABO blood system determines the carbohydrate moieties that are expressed on red blood cells (RBCs) and vascular endothelium. Furthermore, ABO antigens are highly expressed on the surface of a variety of human cells and tissues.

At the beginning of the 20th century, from the time the system was discovered by Karl Landsteiner [1], the ABO blood type was studied widely in relation to diseases and blood transfusion complications. Recent studies have implicated that the ABO blood type is a potential risk for various diseases such as cancer, myocardial infarction, acute kidney injury, and venous thromboembolism [2–5].

Furthermore, the ABO blood type has a profound influence on hemostasis. The occurrence of venous thromboembolism is relatively rare in individuals with blood type O compared with individuals with other blood types [4, 5]. In addition, the relationship between the difference in blood type and the risk of bleeding was reported in patients with several diseases such as upper gastrointestinal hemorrhage [6], hemorrhage during extracorporeal membrane oxygenation therapy [7], and obstetrical hemorrhage [8].

Hemorrhage is the leading cause of death in patients with trauma and could deteriorate the outcome of traumatic brain injury (TBI) due to the increase in intracranial hemorrhage. Therefore, we assessed the association between the difference in blood type and the outcomes of all-cause and cause-specific mortality in patients with severe trauma on the assumption that the outcomes of trauma are affected by the difference in blood type.

## Methods

### Study design and setting

This retrospective observational study aimed to evaluate the association between ABO blood type and mortality in patients with severe trauma who were transported between 1 April 2013 and 31 March 2016 to either of the two tertiary emergency critical care medical centers in Japan (Tokyo Medical and Dental University Hospital of Medicine or Matsudo City Hospital).

This study complied with the principles of the 1964 Declaration of Helsinki and its later amendments. This study was approved by the institutional review board of Tokyo Medical and Dental University and Matsudo City Hospital (#M2017–237 and #29–14, respectively). The requirement for informed consent from each patient was waived because the design of the study was retrospective in nature and because of the use of anonymized patient and hospital data.

### Study populations

We included consecutive patients with trauma who suffered severe injury as defined by an Injury Severity Score (ISS) greater than 15 and who were directly transferred from the scene of injury. Patients who met at least one of the following criteria were excluded from the study: 1) patients younger than 15 years; 2) patients with cardiac arrest upon arrival at the emergency department (ED); 3) patients who suffered unsurvivable injury (Abbreviated Injury Scale (AIS) = 6); and 4) patients with a history of taking anticoagulants or antiplatelet agents. In addition, patients whose clinical data for analysis were missing or insufficient were excluded.

### Data collection

The following information was retrospectively collected from the patients’ medical records: age; sex; blood type (A, B, AB, or O); Charlson Comorbidity Index [9]; the number of patients who received uncrossmatched type O RBC transfusions; AIS [10] of the head and neck, face, chest, abdomen, pelvis and extremities, and surface; ISS; Revised Trauma Score (RTS) calculated based on the Glasgow Coma Scale, systolic blood pressure, and respiratory rate upon arrival at the ED [11]; the total number of units of RBC transfusions administered within 24 h of ED admission; and status upon hospital discharge (i.e., dead or alive). The causes of death were categorized into three groups: exsanguination, TBI, and others (including multiple organ failure). We defined patients with isolated severe TBI as patients with a head AIS ≥ 3 and AIS for other parts of the body ≤ 2.

### Outcomes and definition

The primary outcome was defined as all-cause in-hospital mortality. The secondary outcomes were defined as cause-specific in-hospital mortality (exsanguination, TBI, and others), ventilator-free days (VFD) [12], and total number of units of RBC transfusions administered within the first 24 h from arrival at the ED. One unit of transfusion is prepared from approximately 200 ml of whole blood in Japan, while it is prepared from approximately 450 ml in the USA. Therefore, we prepared transfusion units multiplied by 2.2 to convert to a US standard form and we present them in US units. As previously reported, multiple organ dysfunction syndrome was represented by an increase in the Sequential Organ Failure Assessment score of 2 points or more in more than one organ system [13].

### Statistical analysis

The included population was divided into four groups according to blood type (i.e., A, B, O, and AB). We used a one-way analysis of variance to assess the differences among the four groups. We further evaluated the difference between blood type O and non-O blood type for the outcomes. In univariate analysis, continuous variables were compared using a Student’s *t* test or Mann-Whitney *U* test, and categorical variables were compared using the χ^2^ test or Fisher’s exact test as appropriate. A multivariate logistic or linear regression analysis was performed for the assessment of primary and secondary outcomes after simultaneously controlling for the potential confounders. Variables incorporated into the model were age, RTS, and ISS; these variables were selected based on the clinical perspective (subject matter knowledge) and the number of outcomes (the 10 events per variable rule). The calibration of the model was validated using a Hosmer–Lemeshow goodness-of-fit test; the discrimination of the model was assessed using an optimism-corrected concordance (*c*) statistic, which was calculated in 5000 bootstrap samples. The impact of the difference in blood type on the outcomes was assessed using the value of regression coefficients calculated using a logistic or linear regression analysis and compared with that of other variables.

All statistical analyses were performed using the R software (version 3.4.1; R Foundation for Statistical Computing, Vienna, Austria). A command was used to add statistical functions that were frequently used in the biostatistics. Two-sided *p* values < 0.05 were considered statistically significant.

## Results

**Fig. 1:**
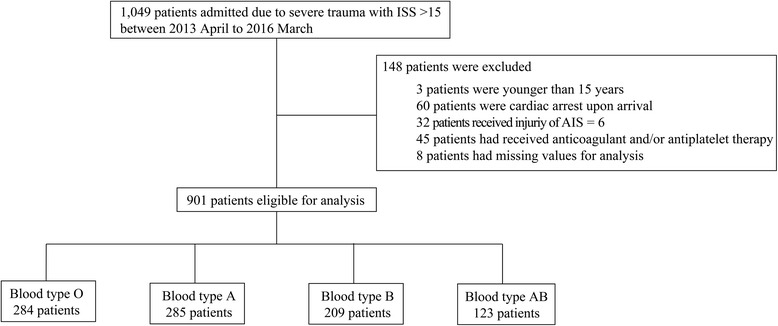
Flow diagram of patient selection. AIS, Abbreviated Injury Scale; ISS, Injury Severity Score

Of 1049 potentially eligible patients, 901 severe trauma patients were included in the analysis (Fig. 1). The study population was divided based on the ABO blood type: type O, 284 (32%); type A, 285 (32%); type B, 209 (23%); and type AB, 123 (13%). The baseline characteristics of patients and outcomes according to blood type are shown in Table 1. Statistically significant difference among the four groups was observed only for RTS (*p* = 0.019); all remaining characteristics were similarly distributed. There was a significant difference in all outcomes except for the number of units of RBC transfusions administered within 24 h. Table 2 provides the comparison of characteristics and the result of univariate analysis for the outcomes between blood type O and other blood types. While there was no significant difference in ISS and AIS in all body regions, patients with blood type O had significantly lower RTS than those with non-O blood type (mean 6.85 (standard deviation (SD) 1.43) in blood type O versus 7.13 (1.15) in other blood types; *p* = 0.003). Although the difference was not statistically significant, the number of units of RBC transfusions administered within 24 h tended to be more in patients with blood type O than those with other blood types (mean 3 (SD 9) units in blood type O versus 2 (7) units in other blood types; *p* = 0.112).

**Table 1:** Baseline characteristics and outcomes in each blood group

	Type O *n* = 284	Type A *n* = 285	Type B *n* = 209	Type AB *n* = 123	*p* value
Characteristics					
Age, years (SD)	57.3 (20.0)	56.3 (20.0)	57.6 (19.4)	53.6 (17.8)	0.284
Female, *n* (%)	64 (22.5)	61 (21.4)	55 (26.3)	26 (21.1)	0.593
Charlson Comorbidity Index, median (IQR)	0 (0–2)	0 (0–2)	0 (0–1)	0 (0–1)	0.380
Uncrossmatched type O RBC transfusion, *n* (%)	28 (9.9)	17 (6)	16 (7.7)	4 (3.3)	0.086
RTS, mean (SD)	6.85 (1.43)	7.12 (1.18)	7.08 (1.16)	7.23 (1.03)	0.019
ISS, median (IQR)	18 (16–25)	18 (16–25)	19 (16–25)	20 (16–25)	0.133
AIS head, median (IQR)	4 (3–4)	4 (3–4)	4 (3–4)	4 (3–4)	0.842
AIS face, median (IQR)	0 (0–0)	0 (0–0)	0 (0–0)	0 (0–0)	0.983
AIS chest, median (IQR)	0 (0–2)	0 (0–3)	0 (0–3)	0 (0–3)	0.612
AIS abdomen, median (IQR)	0 (0–0)	0 (0–0)	0 (0–0)	0 (0–0)	0.781
AIS extremities, median (IQR)	0 (0–2)	0 (0–2)	0 (0–2)	0 (0–2)	0.681
AIS surface, median (IQR)	0 (0–0)	0 (0–0)	0 (0–0)	0 (0–0)	0.873
Outcomes					
In-hospital mortality, *n* (%)	80 (28.2)	30 (10.5)	30 (14.4)	11 (8.9)	< 0.001
Death due to exsanguination, *n* (%)	23 (8.1)	5 (1.8)	5 (2.4)	4 (3.3)	< 0.001
Death due to TBI, *n* (%)	43 (15.1)	22 (7.7)	19 (9.1)	3 (2.4)	< 0.001
Death due to other causes, *n* (%)	14 (4.9)	3 (1.1)	6 (2.9)	4 (3.3)	0.062
Ventilator-free days, mean (SD)	18.7 (12.2)	23.1 (9.3)	22.3 (10.2)	24.5 (8.2)	< 0.001
Units of RBCs administered within 24 h, mean (SD)	3 (11)	2 (5)	2 (10)	2 (8)	0.231

Categorical variables are expressed as numbers (%); continuous variables are presented as medians (25–75 percentiles = interquartile range (IQR))

We used a one-way analysis of variance to assess the differences among the four blood groups

*AIS* Abbreviated Injury Scale; *ISS* Injury Severity Score; *RBC* red blood cell; *RTS* Revised Trauma Score; *SD* standard deviation; *TBI* traumatic brain injury

**Table 2:** The comparison of characteristics and outcomes between blood type O and other blood types

	Type O *n* = 284	Non-O type *n* = 617	*p* value
Characteristics			
Age, years (SD)	57.3 (20.0)	56.2 (19.4)	0.442
Female, *n* (%)	64 (22.5)	142 (23)	0.873
Charlson Comorbidity Index, median (IQR)	0 (0–2)	0 (0–1)	0.829
Uncrossmatched type O RBC transfusion, *n* (%)	28 (9.9)	37 (6)	0.051
RTS, (SD)	6.85 (1.4)	7.13 (1.2)	0.003
ISS, median (IQR)	18 (16–25)	19 (16–25)	0.160
AIS head, median (IQR)	4 (3–4)	4 (3–4)	0.582
AIS face, median (IQR)	0 (0–0)	0 (0–0)	0.783
AIS chest, median (IQR)	0 (0–2)	0 (0–3)	0.342
AIS abdomen, median (IQR)	0 (0–0)	0 (0–0)	0.561
AIS extremities, median (IQR)	0 (0–2)	0 (0–2)	0.443
AIS surface, median (IQR)	0 (0–0)	0 (0–0)	0.652
Outcomes			
In-hospital mortality, *n* (%)	80 (28.2)	71 (11.5)	< 0.001
Death due to exsanguination, *n* (%)	23 (8.1)	15 (2.4)	< 0.001
Death due to TBI, n (%)	43 (15.1)	44 (7.1)	< 0.001
Death due to other causes, *n* (%)	15 (1.8)	11 (5.3)	0.005
Ventilator-free days, mean (SD)	18.7 (12.2)	23.1 (9.4)	< 0.001
Units of RBCs administered within 24 h, mean (SD)	3 (9)	2 (7)	0.112

Categorical variables are expressed as numbers (%); continuous variables are presented as medians (25–75 percentiles = interquartile range (IQR))

*AIS* Abbreviated Injury Scale; *ISS* Injury Severity Score; *RBC* red blood cell; *RTS* Revised Trauma Score; *SD* standard deviation; *TBI* traumatic brain injury

**Fig. 2:**
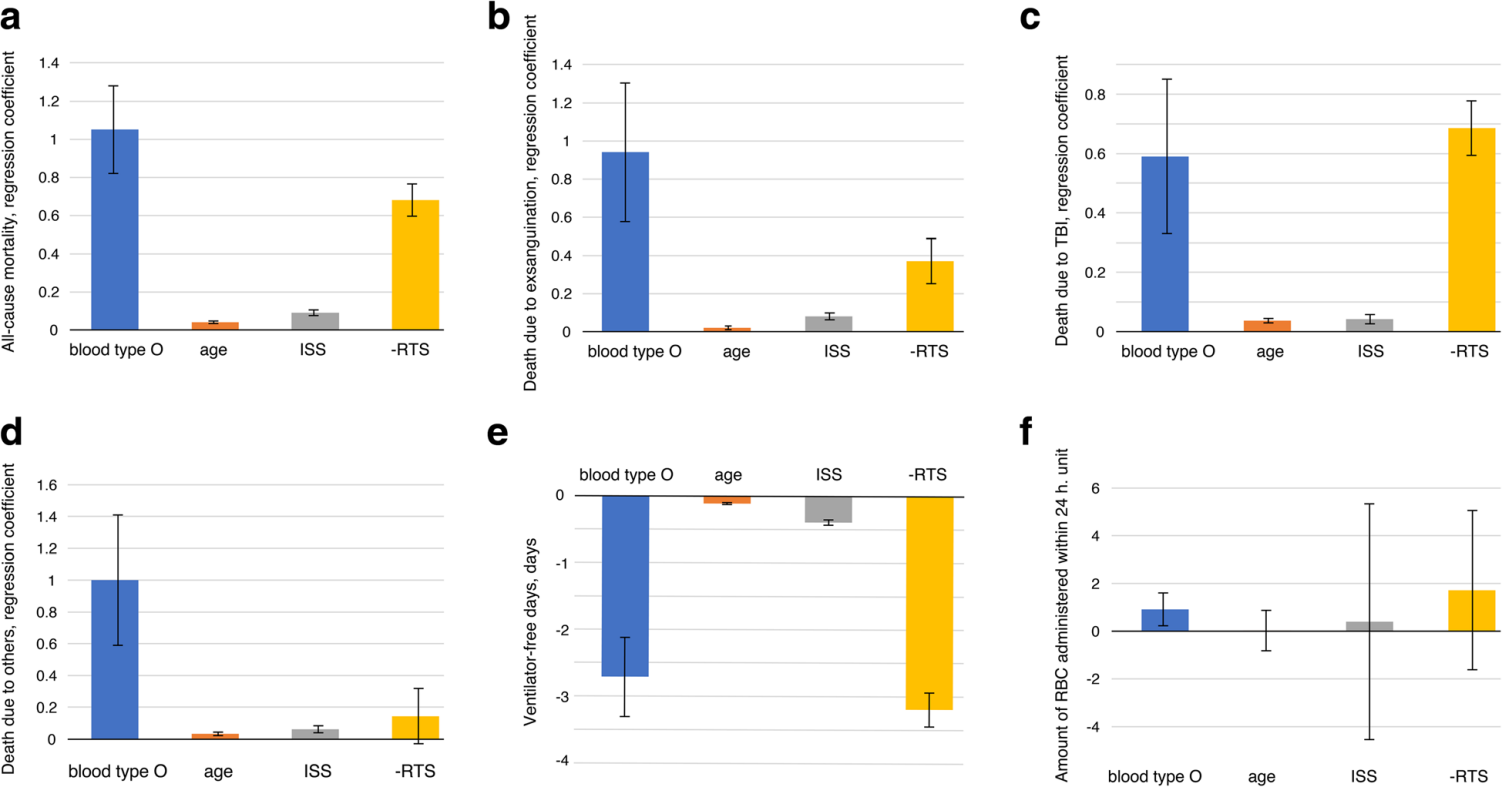
The impact of blood type O and other factors on respective outcome. The bar charts indicate each regression coefficient and the error bars indicate each standard error. ISS, Injury Severity Score; RBC, red blood cell; RTS, Revised Trauma Score; TBI, traumatic brain injury

In the multivariate model, the *p* value of the Hosmer–Lemeshow goodness-of-fit test was 0.269, which suggested that our model had acceptable predictability. The calibration plot is presented in Additional file 1: Figure S1. Furthermore, the value of concordance (*c*) statistics (95% confidence interval (CI)) was 0.88 (0.85–0.91) for crude data, and the value of optimism-corrected *c* statistics calculated in 5000 bootstrapped samples was 0.88. These statistics indicated excellent discrimination for the model, and the issue of overfitting was eliminated in our model. The results of the multivariate analysis are presented in Table 3 and Additional file 2: Table S1. After controlling for age, ISS, and RTS, blood type O continued to be the independent risk factor for all-cause in-hospital mortality (adjusted odds ratio (OR) 2.86 (95% CI 1.84–4.46); *p* <  0.001), death due to exsanguination (adjusted OR 2.55 (95% CI 1.25–5.22); *p* = 0.009), death due to TBI (adjusted OR 1.80 (95% CI 1.08–3.01); *p* = 0.024), death due to other causes (adjusted OR 2.73 (95% CI 1.21–6.13); *p* = 0.015), and shorter VFD (adjusted differences −2.7 days (95% CI −3.9 to −1.6), *p* <  0.001). The impact of blood type O and other factors on respective outcome is shown in Fig. 2.

**Table 3:** Multivariate analysis of the impact of blood type O on outcomes

	Adjusted odds ratio (95% CI)	Adjusted difference (95% CI)	*p* value
Primary outcome			
All-cause in-hospital mortality	2.86 (1.84–4.46)	–	< 0.001
Secondary outcomes			
Death due to exsanguination	2.55 (1.25–5.22)	–	0.009
Death due to TBI	1.80 (1.08–3.01)	–	0.024
Death due to others	2.73 (1.21–6.13)	–	0.015
Ventilator-free days	–	−2.7 (−3.9 to −1.6)	< 0.001
Units of RBCs administered within 24 h	–	1.23 [−0.94–1.36]	0.722

*CI* confidence interval; *RBC* red blood cell; *TBI* traumatic brain injury

The value of the regression coefficient of blood type O for all-cause in-hospital mortality was approximately 12-fold, −1.5-fold, and 26-fold compared with that for ISS, RTS, and age, respectively. This result indicated that the impact of blood type O on in-hospital mortality was comparable to 12 increases in ISS, 1.5 decreases in RTS, and 26 increases in age. For death due to exsanguination, the value of the regression coefficient of blood type O was approximately 12-fold, −2.5-fold, and 55-fold compared with that for ISS, RTS, and age, respectively.

## Discussion

In this retrospective study, we evaluated the association between blood type O and the mortality in 901 patients with severe trauma. Findings indicated that blood type O was the independent risk factor for all-cause in-hospital mortality and death due to exsanguination, TBI, and other causes after adjusting for potential confounders. The number of units of RBC transfusions administered within 24 h upon admission to the ED tended to be more in patients with blood type O; however, there was no significant difference between blood type O and other blood types. To the best of our knowledge, this is the first study to report the association between ABO blood type and mortality in patients with severe trauma. Although we cannot alter the risk of blood type O itself, an adequate recognition of the risk enables us to control for the intensity of trauma critical care. Recognizing those patients requiring damage-control resuscitation is a crucial issue since damage-control resuscitation, including strategic multiple operation, sufficient amounts of available transfusions with adequate ratios, and perioperative critical care, requires a large amount of human and healthcare resources. Therefore, recognizing the additional risk of the difference in blood type would imply the potential to optimize the implementation or damage-control resuscitation and indirectly improve the outcome of patients with severe trauma.

Several studies have reported that patients with blood type O had 25–30% lower plasma von Willebrand factor (vWF) levels than the those with non-O blood, increasing their risk of hemorrhage [14, 15]. vWF plays a decisive role in primary hemostasis by mediating the adhesion of blood platelets to the subendothelium of the damaged vessel walls and promoting the aggregation of activated platelets. In addition, vWF acts as a carrier of factor VIII clotting activity and protects it from premature proteolysis [16–18]. Therefore, a lower vWF level is a possible explanation for the increase in mortality in patients with blood type O, as observed in this study. However, many of the differences in the mechanisms of hemostasis according to blood type remain unknown. Further basic study is warranted to reveal the role of blood type in maintaining hemostasis during critical situations.

Although the results of the multivariate analysis showed that blood type O was an independent risk factor for all-cause trauma-related mortality and death due to exsanguination, there was no significant difference in the number of units of RBC transfusions administered within 24 h of admission in patients with blood type O compared with those with other blood types. One possible explanation for this discrepancy is the issue of survivor bias. Patients who died shortly after ED arrival or who did not have a surgical indication due to too severe an injury were less likely to receive large amounts of transfusion. Another possible explanation was beta error. The number of patients who received a RBC transfusion was only 20% of the total patients included, and it prevented us from detecting the significant difference between the two groups.

In this study, although ISS was similar between the two groups, RTS observed in patients with blood type O was lower than that in patients with other blood types. The physiological status of patients with blood type O may deteriorate even if these patients suffered injuries of the same intensity. Because this was a retrospective cohort study with a limited sample size, patient background could not be similarly distributed, and it was uncertain whether the statistically significant difference observed in RTS was a clinically meaningful result. Although completely adjusting the difference using a statistical approach was generally insufficient, in this study the RTS value was incorporated into the model and the difference was accounted for. Our results demonstrated that the impact of blood type O on trauma death was extremely high. The impact of blood type O on all-cause mortality was comparable to 12 increases in ISS, 1.5 decrease in RTS, and 26 increases in age. Furthermore, the impact of blood type O seemed to be higher for the outcome of death due to exsanguination than the all-cause trauma-related mortality. It would be of great significance for clinicians to recognize the potential risk of blood type O.

Several limitations should be considered in the interpretation of our findings. First, because this was a retrospective study with a limited sample size, the risk of residual confounding and the risk of type I error remain. Additional work is necessary to provide more definitive data, including large-scale multicenter studies. Second, we only evaluated the phenotype and not the genotype of the ABO blood group or Rh system. The highest levels of clotting vVIII and vWF were observed in the A_1_A/A_1_B/BB genotype, intermediate levels in the A_1_O/BO genotype, and lowest levels in the OO genotype [19]. Possibly, by comparing type O to non-O blood type instead of A_1_A/A_1_B/BB to OO, the effect of ABO on prognosis was diluted. In addition, in this study, only two patients had Rh-negative blood, and this prevented us from assessing the statistical difference between Rh-positive and Rh-negative patients. Third, all the patients analyzed in this study were Japanese; therefore, it is unclear whether our findings apply to other ethnic groups. Finally, patients who had received anticoagulant and/or antiplatelet therapy were excluded from this study because these agents could largely influence the mortality and hemorrhage in severe trauma patients; this process could cause a selection bias—if the difference in blood type is related to thrombotic diseases, patients with the blood type that were less likely to suffer from thrombotic diseases were less likely to receive those therapies. However, in this study, the excluded patients due to anticoagulant and/or antiplatelet therapy was similarly distributed among each blood type (type O, 12; type A, 15; type B, 10; and type AB, 8); therefore, this process would not largely affect the results. Despite these limitations, we initially show a significant association between blood type O and mortality in patients with severe trauma. Furthermore, the impact of blood type O on trauma-related death was found to be high. Further research beyond this epidemiological study that evaluates where and how ABO antigens work (through blood, endothelial, or vWF, and the mechanisms, respectively) are warranted to assess the effects of emergency uncrossmatched type O RBC transfusion and the possibility of novel therapeutic interventions.

## Conclusion

The results of this study suggest that blood type O is associated with mortality and exsanguination in patients with severe trauma. Further basic or translational research is necessary to investigate the cause of this result and may lead to the development of a therapeutic intervention.

## Additional files

Additional file 1: **Figure S1.** The diagonal line represents the line of perfect fit between observed and predicted risk. (TIFF 2540 kb)

Additional file 2: **Table S1.** Multivariate analysis of the factors influencing outcomes and the comparison of explanatory variables. (DOCX 72 kb)

## Abbreviations

AIS: Abbreviated Injury Scale
CI: Confidence interval
ED: Emergency department
ISS: Injury Severity Score
OR: Odds ratio
RBC: Red blood cell
RTS: Revised Trauma Score
SD: Standard deviation
TBI: Traumatic brain injury
VFD: Ventilator-free days
vWF: Von Willebrand factor
