# Supplementary figures and images for: The impact of blood type O on mortality of severe trauma patients: a retrospective observational study
Source: Crit Care. 2018 May 2;22:100. doi: 10.1186/s13054-018-2022-0 (PMC5930809; doi:10.1186/s13054-018-2022-0)

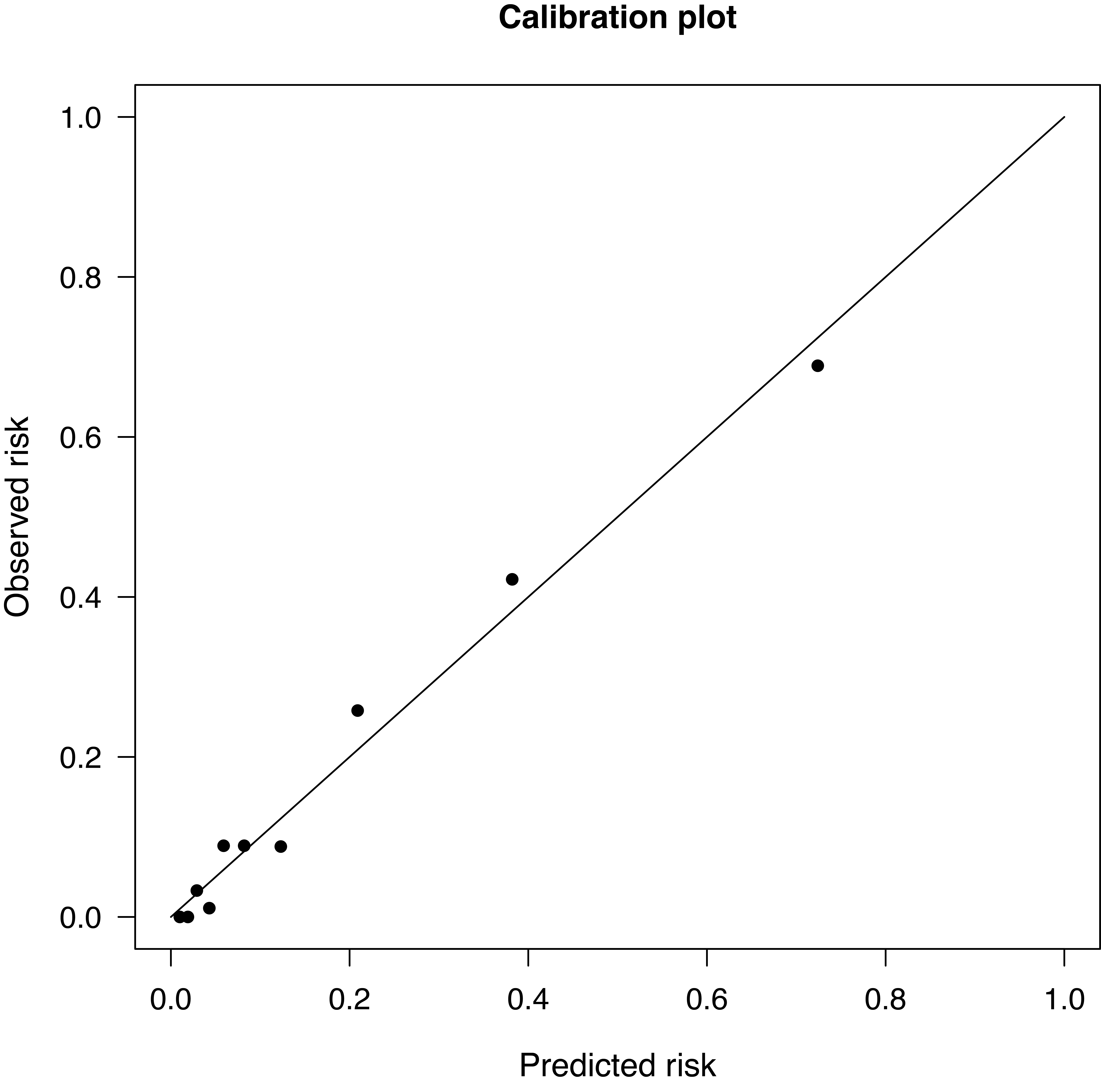

Supplement: Supplementary file 1 — Figure S1. The diagonal line represents the line of perfect fit between observed and predicted risk. (TIFF 2540 kb) [file 13054_2018_2022_MOESM1_ESM.tif]
