# Supplementary material for: The impact of blood type O on mortality of severe trauma patients: a retrospective observational study
Source: Crit Care. 2018 May 2;22:100. doi: 10.1186/s13054-018-2022-0 (PMC5930809; doi:10.1186/s13054-018-2022-0)
Supplement: Supplementary file 2 — Table S1. Multivariate analysis of the factors influencing outcomes and the comparison of explanatory variables. (DOCX 72 kb) [file 13054_2018_2022_MOESM2_ESM.docx]

| **Table S1: Multivariate analysis of the factors influencing outcomes and the comparison of explanatory variables** | | | | | | | | | | | | | | |
| --- | --- | --- | --- | --- | --- | --- | --- | --- | --- | --- | --- | --- | --- | --- |
|  | | | Regression coefficient | | | Adjusted odds ratio [95% CI] | | | Adjusted difference  [95% CI] | | | *p* value | | |
| **Primary outcome** | | | | | | | | | | | | | | |
|  | All-cause in-hospital mortality | | | | | | | | | | | | | |
|  |  | Blood type O | 1.05 | | | 2.86 [1.84–4.46] | | | – | | | <0.001 | | |
|  |  | Age | 0.04 | | | 1.04 [1.03–1.06] | | | – | | | <0.001 | | |
|  |  | ISS | 0.09 | | | 1.10 [1.07–1.13] | | | – | | | <0.001 | | |
|  |  | RTS | -0.68 | | | 0.51 [0.43–0.60] | | | – | | | <0.001 | | |
| **Secondary outcomes** | | | | | | | | | | | | | | |
|  | Death due to exsanguination | | | | | | | | | | | | | |
|  |  | Blood type O | 0.94 | | | 2.55 [1.25–5.22] | | | – | | | 0.009 | | |
|  |  | Age | 0.02 | | | 1.02 [0.99–1.04] | | | – | | | 0.072 | | |
|  |  | ISS | 0.08 | | | 1.08 [1.04–1.12] | | | – | | | <0.001 | | |
|  |  | RTS | -0.37 | | | 0.69 [0.55–0.87] | | | – | | | 0.001 | | |
|  | Death due to TBI | | | | | | | | | | | | | |
|  |  | Blood type O | | | 0.59 | | | 1.80 [1.08–3.01] | | | – | | | 0.024 |
|  |  | Age | | | 0.04 | | | 1.04 [1.02–1.05] | | | – | | | <0.001 |
|  |  | ISS | | | 0.04 | | | 1.04 [1.01–1.08] | | | – | | | 0.007 |
|  |  | RTS | | | -0.69 | | | 0.50 [0.42–0.60] | | | – | | | <0.001 |
|  | Death due to others | | | | | | | | | | | | | |
|  |  | Blood type O | | 1.00 | | | 2.73 [1.21–6.13] | | | – | | | 0.015 | |
|  |  | Age | | 0.03 | | | 1.04 [1.01–1.06] | | | – | | | 0.003 | |
|  |  | ISS | | 0.06 | | | 1.07 [1.02–1.11] | | | – | | | 0.005 | |
|  |  | RTS | | 0.15 | | | 1.16 [0.82–1.63] | | | – | | | 0.398 | |
|  | Ventilator-free days | | | | | | | | | | | | | |
|  |  | Blood type O | | -2.71 | | | - | | | -2.7 [-3.9–-1.6] | | | < 0.001 | |
|  |  | Age | | -0.11 | | | - | | | -0.1 [-0.2–-0.1] | | | < 0.001 | |
|  |  | ISS | | -0.39 | | | - | | | -0.4 [-0.5 –-0.3] | | | < 0.001 | |
|  |  | RTS | | 3.19 | | | - | | | 3.2 [2.7 –3.7] | | | < 0.001 | |
|  | Amount of RBC administered within 24 h | | | | | | | | | | | | | |
|  |  | Blood type O | | 0.21 | | | – | | | 1.23 [-0.94–1.36] | | | 0.722 | |
|  |  | Age | | -0.02 | | | – | | | -0.02 [-0.05–0.08] | | | 0.163 | |
|  |  | ISS | | 0.32 | | | – | | | 0.32 [0.25–0.40] | | | <0.001 | |
|  |  | RTS | | -0.65 | | | – | | | -0.65 [-1.15–-0.16] | | | 0.009 | |
| Abbreviations: SD, standard deviation; RTS, Revised Trauma Score; ISS, Injury Severity Score; TBI, traumatic brain injury; RBC, red blood cell | | | | | | | | | | | | | | |
